# Quality of life and the associated factors among postmenopausal women during the COVID-19 pandemic: a cross-sectional study

**DOI:** 10.1007/s00737-025-01581-2

**Published:** 2025-04-03

**Authors:** Sakda Arj-Ong Vallibhakara, Nichakorn Piyatham, Orawin Vallibhakara, Jittima Manonai

**Affiliations:** 1 Interdisciplinary Studies and Lifelong Education, Faculty of Public Health, Mahidol University, Bangkok, Thailand; 2 Department of Obstetrics and Gynecology, Faculty of Medicine, Ramathibodi Hospital, Mahidol University, Bangkok, Thailand; 3 Menopause Unit, Reproductive Endocrinology and Infertility Unit, Department of Obstetrics and Gynaecology, Faculty of Medicine, Ramathibodi Hospital, Mahidol University, Bangkok, Thailand; 4 Female Pelvic Medicine and Reconstructive Surgery Unit, Department of Obstetrics & Gynaecology, Faculty of Medicine, Ramathibodi Hospital, Mahidol University, Bangkok, Thailand

**Keywords:** Menopause, Quality of life, Menopausal symptoms, COVID-19, Well-being

## Abstract

**Purpose:**

To evaluate the quality of life (QOL) of postmenopausal women during the COVID-19 pandemic in Thailand and the associated factors.

**Methods:**

A cross-sectional study using a validated Thai version of the WHOQOL-BREF questionnaire to assess QOL was conducted.

**Results:**

A total of 453 postmenopausal women participated in the study, with a median age of 58 years (ranging from 41 to 65). These women, residing in urban areas of Thailand such as Bangkok, Nonthaburi, Nakhon Pathom, Pathum Thani, Samut Prakan, and Samut Sakhon, completed a survey questionnaire with the assistance of health volunteers during the COVID-19 pandemic, between October 2021 and April 2022. The study revealed that 55.85% of participants reported experiencing poor quality of life (QOL) during the pandemic. Stepwise logistic regression analysis identified several significant associations with QOL. Factors linked to a poorer QOL included lower levels of well-being index (odds ratio [OR] 7.89, 95% confidence interval [CI] 3.16–19.75, *P* < 0.01), reduced physical activity (OR 2.72, 95% CI 1.62–4.54, *P* < 0.01), and the severity of menopausal symptoms (OR 1.94, 95% CI 1.42–2.66, *P* < 0.01). Conversely, higher education (OR 0.28, 95% CI 0.10–0.75, *P* < 0.01), an earlier onset of menopause (OR 0.55, 95% CI 0.39–0.78, *P* < 0.01), and occasional social alcohol consumption (OR 0.56, 95% CI 0.33–0.94, *P* = 0.03) were identified as protective factors for QOL.

**Conclusion:**

These findings highlight the importance of comprehensive menopausal care, addressing both physical and mental well-being, and providing specific care for menopausal symptoms during challenging times of the COVID-19 pandemic.

## Introduction

Menopause is a key stage in women’s reproductive health, characterized by a decline in estrogen levels that can negatively affect quality of life (QOL). Common short-term effects include hot flashes, night sweats, insomnia, mood changes, anxiety, and genitourinary syndrome of menopause (GSM). Long-term, postmenopausal women may face increased risks of cardiovascular disease, cognitive decline, and musculoskeletal aging (Santoro et al. 2015). These menopause-related changes can be distressing and serve as significant determinants of reduced quality of life in women (Monteleone et al. 2018). Panichkul S et al. reported in 2017 that the prevalence of impaired quality of life among menopausal women in Central Thailand was around 47.4% (Panichkul et al. 2017). These changes have coincided with the COVID-19 pandemic, which was declared by the World Health Organization (WHO) on March 11, 2020. COVID-19 spreads through respiratory droplets and can cause symptoms like fever, cough, and loss of smell. Older adults and those with preexisting conditions are particularly vulnerable to severe illness and death. Since its emergence in December 2019 in Wuhan, China, COVID-19 has affected over 200 million people globally, resulting in more than 6 million deaths by August 9, 2021. Consequently, many countries implemented preventive measures, such as social distancing, lockdowns, and vaccination were emphasized to combat the spread of the virus (Priyadarshini et al. 2020; Cucinotta et al. 2020).

Thailand reported its first COVID-19 case on January 13, 2020, leading to its first wave of infections linked to a Thai boxing stadium and returning tourists. A state of emergency was declared on March 25, 2020, followed by a lockdown starting April 4, which included a ban on commercial flights until July 1. Preventive measures like work-from-home arrangements, online learning, and restricted travel helped reduce cases by May 2020, but subsequent infection waves occurred in December 2020 and March to April 2021, with vaccinations beginning in late February 2021 (Srichannil et al. 2020; Marome et al. 2021). Access to healthcare became challenging for postmenopausal women during the pandemic due to lockdowns, further worsening menopausal symptoms and affecting their quality of life (QOL). The World Health Organization defines QOL as an individual’s perception of their life circumstances, including culture, values, goals, expectations, standards, and concerns. It aligns with the Sustainable Development Goals (SDG) 2030, particularly the “Establishing Good Health and Well-Being agenda.” and well-being, aligning with the Sustainable Development Goals (SDGs) 2030. Factors influencing the QOL of postmenopausal women include physical health, lifestyle, emotional well-being, and social conditions. Women over 60 are at high risk for severe COVID-19 symptoms due to age-related physiological changes (Whitley et al. 2013; Nazarpour et al. 2020). Overall, there is limited information on the QOL of postmenopausal women during the pandemic, particularly in Thailand.

## Materials and methods

### Study designs

We conducted a cross-sectional study to explore the effect of the COVID-19 pandemic on the quality of life among menopausal women in urban Thailand, Bangkok, and its surrounding areas, including Nakhon Pathom, Nonthaburi, Pathum Thani, Samut Prakan, and Samut Sakhon at the end of the third wave pandemic (at the end of 2021). The primary outcome was the prevalence of postmenopausal women with poor quality of life (QOL) during the COVID-19 pandemic. The secondary results were the factors associated with QOL during the widespread outbreak of COVID-19. The study was approved by the Ethical Clearance Committee on Human Rights Related to Research Involving Human Subjects Faculty of Medicine Ramathibodi Hospital, Mahidol University COA. MURA2021/844 (date of approval October 06, 2021). The data collection was performed after ethical approval.

### Participants

The study enrolled a cohort of healthy postmenopausal women aged between 41 and 65 years, residing in urban areas of Thailand, including Bangkok, Nonthaburi, Nakhon Pathom, Pathum Thani, Samut Prakan, and Samut Sakhon, during the COVID-19 outbreak period from 20 October 2021 to 30 April 2022. All participants provided informed consent before participation. The inclusion criteria comprised menopausal women with a history of amenorrhea for at least one year or those who had undergone bilateral oophorectomy or exhibited serum FSH levels greater than 40 IU/L. Proficiency in reading and understanding the Thai language was also a requirement for inclusion. Conversely, individuals currently undergoing treatment for COVID-19, those with a history of cancer or psychiatric disorders, and those with severe medical conditions like cardiovascular disease, coronary heart disease, heart failure, liver failure, and renal failure were excluded from the study. The sample size was determined using the infinite population proportion formula, based on previous research findings by Zhang Y et al. (Zhang Y et al. 2020) with an α-error of 0.05. The calculated sample size was 383, and after accounting for a 10% data loss, the overall sample size was adjusted to 420.

### Data collection and measurements

After obtaining informed consent, participants were asked to complete a comprehensive questionnaire facilitated by the research team, which included a doctor, nurse, and staff. This process typically took 20 to 30 min. The questionnaire consisted of four parts: baseline characteristics, quality of life and well-being, physical activity behaviour, and mental health. Baseline characteristics included age, age at menopause, menopausal symptoms, parity, medical history, current medication, smoking and drinking history, exercise habits, sexual activity, socioeconomic status, and education level. Additionally, data on the impact of the COVID-19 pandemic, including socioeconomic status, family history of COVID-19 infection, and COVID-19 vaccination status, were collected.

Quality of life (QOL) evaluation was conducted using the validated Thai version of the World Health Organization Quality of Life Brief (WHOQOL-BREF-THAI) questionnaire. The QOL evaluation in this study utilized a validated Thai language questionnaire known as WHOQOL-BREF-THAI. This questionnaire demonstrated excellent validity, with a content validity of 0.65 and high reliability, as indicated by a Cronbach’s alpha coefficient of 0.84 (Department of Mental Health 2016). This 26-item self-report questionnaire assesses QOL across four domains: Physical health (7 items), Psychological (6 items), Social Relationships (3 items), Environment (8 items), and overall health (2 items). Each item is rated on a 5-point estimation scale comprising 23 positive and three negative questions. The total scores range from 26 to 130, with higher scores indicating a better quality of life. Interpretation of scores classifies QOL as poor (26–60), moderate (61–95), or good (96–130).

Physical activity encompasses any bodily movement that requires energy and engages muscles, including activities related to work, travel, and recreation. It is categorized into two types: moderate and vigorous physical activity. Adequate physical activity, as defined by the World Health Organization (WHO), involves engaging in at least 150 min of moderate physical activity per week, 75 min per week of vigorous physical activity, or a combination of both. The Global Physical Activity Questionnaire (GPAQ Version 2) was employed in this study to assess physical activity levels, which has been appropriately translated into Thai. The GPAQ consists of 16 questions covering three types of physical activity: work-related, travel between places, and leisure-time activities. The internal consistency of the GPAQ, measured by Cronbach’s alpha coefficient, was found to be 0.83 (Jaturapatporn et al. 2006). The GPAQ results were converted into metabolic equivalent minutes (MET) per week to quantify the total energy expenditure per week. This calculation is based on the duration of moderate activity minutes per week multiplied by four and the number of vigorous activity minutes multiplied by eight. Adequate physical activity was determined by achieving a GPAQ score of at least 600 min per week, while a GPAQ score below 600 min per week indicated insufficient physical activity.

The Menopause Rating Scale (MRS) is utilized to assess menopausal symptoms by evaluating the severity of symptoms across three categories: somatic (4 items), psychological (4 items), and urogenital (3 items). Each symptom is rated on a scale from 0 to 4, with the meanings as follows: 4 indicates “very severe,” 3 means “severe,” 2 represents “moderate,” 1 signifies “mild,” and 0 denotes “no symptoms.” A total MRS score of 16 or higher suggests severe symptoms and may necessitate a referral to a gynecologist (Heinemann el al. 2004) The Thai version of the MRS, evaluated by Phanucharas D in 2013, demonstrated a sensitivity of 69.3% and a specificity of 76.9% for screening purposes (Phanucharas 2013).

The study evaluated mental health using the World Health Organization’s Happiness Index (WHO-Five Well-Being Index, Thai version) and the Generalized Anxiety Disorder (GAD-7) questionnaire. The WHO-Five Well-Being Index (Thai version) questionnaire (Saipanish et al. 2009) yields scores ranging from 0 to 25, where 0 signifies the least likelihood of having a good quality of life, and 25 indicates the highest likelihood of having a good quality of life. On the other hand, GAD-7 scores between 0 and 9 indicate average or slightly higher-than-average levels of anxiety, scores between 10 and 14 indicate moderate anxiety, and scores between 15 and 21 indicate high anxiety levels (RamaMental 2015).

### Statistical analysis

Descriptive data were presented for baseline characteristics. Normality testing was conducted for all quantitative variables, and accordingly, mean (standard deviation, S.D.) was used for normally distributed data, while median (range) was utilized for non-normally distributed data. STATA Version 16.0 (College Station, TX, USA) performed the statistical analysis. Univariate analysis was initially employed to identify factors associated with poor quality of life (QOL). Factors showing a significant association (p-value < 0.10) in the univariate analysis were then subjected to multiple logistic regression to assess their independent effects. A p-value < 0.05 was considered for statistical significance, and 95% confidence intervals (CI) were reported.

## Results

A total of 480 participants were invited to participate in the study, resulting in a response rate of 95%. The complete questionnaire rate was 99%, with 453 participants manually completing the survey questionnaire, which took approximately 20–30 min. Health volunteers, researchers, and nurses guided participants in cases of uncertainty or difficulty understanding the questions. The prevalence of poor quality of life (QOL) among menopausal women was found to be 55.85%, with 253 out of 453 participants. The baseline characteristics of the participants revealed a median age of 58 years (ranging from 41 to 65) and a median body mass index (BMI) of 23.87 kg/m² (ranging from 15.5 to 38.4). Most participants experienced natural menopause, with a small proportion having a history of menopausal hormone therapy use (11.92%). Approximately half of the participants reported moderate to severe menopausal symptoms, as evaluated by the menopausal rating scale (MRS). The educational background showed that one-third of the participants held Bachelor’s degrees (36.2%), and nearly one-third were engaged in full-time jobs with fixed incomes (31.79%). The median monthly income was 30,000 Thai baths (approximately 873 USD), primarily reflecting middle- to high-income families. Most participants were married but sexually inactive, had one or two children, resided in Bangkok, and owned a house, see Tables 1 and 2. Regarding the COVID-19 situation, most participants had received the COVID-19 vaccine (85.9%), with most having completed both doses. A few participants had experienced COVID-19 infection (4.86%), but approximately half reported being affected by the COVID-19 outbreak, 223 cases (49.23%), as indicated in Table 3.

**Table 1:** Socioeconomic baseline characteristics

Characteristics	*N* = 453 (%)
Age*	58 (41, 65)
Status	
Single	74 (16.34)
Married	292 (64.46)
Divorce or widow	87 (19.21)
Habitat	
Own house	378 (83.44)
Rental or others	75 (16.56)
Education	
Below Bachelor’s degree	239 (52.76)
Bachelor’s degree	164 (36.20)
Above Bachelor’s degree	50 (11.04)
Occupation	
No salary	217 (47.90)
Fixed income (salary)	144 (31.79)
Not fixed income	92 (20.31)
Income (USD)*	873 (0, 5,820)
Alcohol	
No	424 (93.60)
Drinking	29 (6.40)
Smoking	
No smoking	445 (98.23)
Smoking	8 (1.77)

Data shown as N (%), and * median (min, max)

Abbreviation: USD US Dollars

**Table 2:** Physical health and menopausal status baseline

Characteristics	*N* = 453 (%)
BMI (kg/m^2^) *	23.87 (15.5, 38.4)
Menopause age*	50 (29, 59)
Underlying	
No underlying disease	203 (44.81)
Had underlying disease	250 (55.19)
Parity	
0	120 (26.49)
1–2 persons	284 (62.69)
More than3 persons	49 (10.82)
Hormonal replacement therapy	
None	399 (88.08)
Use	54 (11.92)
Total Menopausal Rating scale	
0–4 (none)	105 (23.18)
5–8 (mild)	101 (22.30)
9–15 (Moderate)	138 (30.46)
More than 15 (severe symptoms)	109 (24.06)
Active sexual intercourse	
No	319 (70.00)
Yes	134 (30.00)

Data shown as N (%), and * median (min, max)

The underlying disease including type 2 diabetes mellitus, hypertension and dyslipidemia

**Table 3:** Baseline COVID-19 infection and vaccination

Characteristics	*N* = 453 (%)
COVID-19 infection	
Never	431 (95.14)
Previous infection	22 (4.86)
Effect of COVID-19 on Quality of Life	
No	230 (50.77)
Yes	223 (49.23)
COVID-19 vaccination	
No	12 (2.65)
Yes	441 (97.35)
Number of vaccine doses	
1	13 (2.95)
2	379 (85.94)
3	45 (10.20)
4	4 (0.91)

Data are shown as N (%)

The prevalence of poor quality of life (QOL) among menopausal women during the COVID-19 pandemic was 55.85%, defined by WHOQOL-BREF-THAI scores below 95 points. Furthermore, approximately two-thirds of the participants exhibited low physical activity levels across work, travel, and leisure activities. However, most participants reported good mental health, as evidenced by positive well-being index scores and normal average anxiety levels, as presented in Table 4.

**Table 4:** Quality of life, physical activity, well-being, and level of anxiety among postmenopausal women

Characteristics	*N* = 453 (%)
Quality of life	
Good QOL	200 (44.15)
Poor QOL	253 (55.85)
GPAQ for working (MET-minutes)	
≥ 600	169 (37.30)
< 600	284 (62.70)
GPAQ for leisure activity (MET-minutes)	
≥ 600	134 (29.58)
< 600	319 (70.42)
Well-Being Index	
≥13 Good well-being	370 (81.68)
<13 Not well-being	83 (18.32)
GAD-7	
0–9 (Average level of anxiety)	406 (89.62)
10–14 (Moderate level of anxiety)	36 (7.95)
15–21 (High level of anxiety)	11 (2.43)

Abbreviation: QOL quality of life, GPAQ The Global Physical Activity Questionnaire, MET metabolic equivalent minutes, GAD Generalized Anxiety Disorder

Our results from univariate analysis showed factors associated with poor QOL among postmenopausal women during the pandemic included low education level, low income (less than 35,000 THB per month or 873 USD), multiparity, and the presence of medical illnesses. Additionally, menopausal age after 45 and moderate to severe vasomotor symptoms were significant factors linked to poor QOL. Obesity (BMI > 30 kg/m²), low levels of physical activity in various domains (work, travel, and leisure activities), low well-being index scores, self-reported impact due to the COVID-19 situation, and high anxiety scores were also significantly associated with poor QOL. However, a history of COVID-19 infection, infection among family members, or vaccination did not significantly impact QOL as demonstrated in Table 5. These findings shed light on the complex interplay of various factors influencing the QOL of postmenopausal women during the COVID-19 pandemic, offering valuable insights for targeted interventions and support strategies.

**Table 5:** Factor associated with poor quality of life in postmenopausal women during the COVID-19 pandemic -univariate analysis

Factors	Good QOL (*N* = 200)	Poor QOL (*N* = 253)	*P* value
Status			0.102
Single	41 (55.41)	33 (44.59)	
Married	122 (41.78)	170 (58.22)	
Divorce or widow	37 (42.53)	50 (57.47)	
Educate			0.000 *
Below Bachelor’s degree	86 (35.98)	153 (64.02)	
Bachelor’s degree	82 (50.00)	82 (50.00)	
Above Bachelor’s degree	32 (64.00)	18 (36.00)	
Occupation			0.001 *
no salary	115 (53.00)	102 (47.00)	
salary	53 (36.81)	91 (63.19)	
no constant income	32 (34.78)	60 (65.22)	
Income			0.046 *
> 35,000 baths	97 (49.49)	99 (50.51)	
≤ 35,000 baths	103 (40.08)	154 (59.92)	
BMI			0.044 *
BMI < 30 kg/m^2^	188 (45.63)	224 (54.37)	
BMI ≥ 30 kg/m^2^	12 (29.27)	29 (70.73)	
Menopause age			0.006 *
> 45 years old	141 (40.63)	206 (59.37)	
≤ 45 years old	59 (55.66)	47 (44.34)	
Underlying medical disease			0.011 *
no	103 (50.74)	100 (49.26)	
yes	97 (38.80)	153 (61.20)	
Alcohol drinking			0.017 *
no	181 (42.69)	243 (57.31)	
yes	19 (65.52)	10 (34.48)	
Parity			0.086 *
0–1	61 (50.83)	59 (49.17)	
More than 1	139 (41.74)	194 (58.26)	
Total Menopausal Rating scale			0.000 *
0–4 (none)	73 (69.52)	32 (30.48)	
5–8 (mild)	58 (57.43)	43 (42.57)	
9–15 (Moderate)	47 (34.06)	91 (65.94)	
More than 15 (severe symptoms)	22 (20.18)	87 (79.82)	
Anxiety score (GAD-7 score)			< 0.0001*
Average level of anxiety (0–9)	191 (47.04)	215 (52.96)	
Moderate to high level of anxiety (10–21)	9 (19.15)	38 (80.85)	
Active sexual intercourse			0.426
no	137 (42.95)	182 (57.05)	
yes	63 (47.01)	71 (52.99)	
Covid-19 infection			0.102
Never	194 (45.01)	237 (54.99)	
Previous infection	6 (27.27)	16 (72.73)	
Affected by the COVID-19 Outbreak.			0.003 *
No	117 (50.87)	113 (49.13)	
Yes	83 (37.22)	140 (62.78)	
COVID-19 vaccination			0.176
No	3 (25.00)	9 (75.00)	
Yes	197 (44.67)	224 (55.33)	
Number of vaccine doses			0.006 *
1	4 (30.77)	9 (69.23)	
2	161 (42.48)	218 (57.52)	
3	28 (62.22)	17 (37.78)	
4	4 (100.00)	0 (0.00)	

*Statistically significant (p-value < 0.10) before analyses of multiple logistic regression

Abbreviation: BMI body mass index, GAD Generalized Anxiety Disorder

After performing the stepwise logistic regression analysis, we identified the most significant factors associated with poor quality of life (QOL) among menopausal women during the COVID-19 pandemic. Low well-being or happiness scores emerged as the strongest predictor (OR = 7.89, 95%CI 3.16–19.75, *P* < 0.01), followed by inadequate leisure time (OR = 2.72, 95%CI 1.62–4.54, *P* < 0.01), and the presence of moderate to severe menopause symptoms (OR = 1.94, 95%CI 1.42–2.66, *P* < 0.01).

Conversely, certain factors showed a protective effect on QOL. Occasional social alcohol consumption (OR 0.28, 95% CI 0.10–0.75, *P* = 0.012), early menopause before the age of 45 (OR  =  0.56, 95%CI 0.33–0.94, *P* =  0.028), and higher education level (OR = 0.55, 95%CI 0.39–0.78, *P* =  0.001 = 0.28, 95%CI 0.10–0.75, *P* = 0.01) were statistically significant factors associated with better QOL. The findings, detailed in Table 6, underscore the importance of addressing psychosocial and lifestyle factors to enhance the QOL of menopausal women during the challenging circumstances of the COVID-19 pandemic.

**Table 6:** Significant factors associated with poor quality of life in postmenopausal women during the COVID-19 Pandemic - multivariate analysis

Factors	Odds Ratio (OR)	*P* value*	95% confidence interval
Low WHO-Five Well-Being Index	7.89	< 0.001	3.16–19.75
Low Physical activity for leisure activity	2.72	< 0.001	1.62–4.54
Menopause Rating Scale (Exhausted)	1.94	< 0.001	1.42–2.66
Menopause Rating Scale (Joint pain)	1.49	0.005	1.13–1.98
Early menopausal age	0.56	0.028	0.33–0.94
Higher education	0.55	0.001	0.39–0.78
Occasional social alcohol consumption	0.28	0.012	0.10–0.75

*Statistically significant (p-value < 0.05)

## Discussion

To explore the prevalence of poor QOL and the associated factors among menopausal women during COVID-19 pandemic. Menopausal women face unique challenges to their quality of life, especially during exceptional circumstances like the COVID-19 pandemic, which can significantly impact their well-being (Nazarpour et al. 2020; Schneider et al. 2017). Prior research has explored the prevalence of impaired quality of life in peri- and postmenopausal women. For instance, Panichkul et al. reported a prevalence of 28.2% among Thai peri- and postmenopausal women using the Menopause-Specific Quality of Life Questionnaire (MENOQOL). Younger women experienced significantly more impaired quality of life. The most common menopause-related symptoms were psycho-emotional distress, musculoskeletal pain, and hot flashes. Interestingly, all quality-of-life scores improved over time, regardless of the use of postmenopausal hormone therapy (Panichkul et al. 2017). Similarly, Krishnamoorthy et al. found a prevalence of 37.2% poor quality of life among postmenopausal women in South India, as assessed by WHOQOL-BREF. Factors associated with poor quality of life included living in a nuclear family, having underlying medical conditions, and using alcohol or tobacco. One-third of women with poor quality of life reported psychological problems (Krishnamoorthy et al. 2018). Additionally, Çalişkan Pala et al. discovered a 23% prevalence of depression in a survey of menopausal women in a semi-rural area in Turkey, with a moderate negative correlation between the quality of life and depression (Çalişkan Pala et al. 2022).

In addition to the challenges posed by the menopausal transition, the global COVID-19 pandemic since 2019 has significantly impacted various aspects of life, including individual well-being, social interactions, environmental factors, economic stability, and public health. The COVID-19 control measures, such as social distancing, isolation, city lockdowns, restrictions, and personal hygiene practices, have had direct and indirect effects on the quality of life (QOL) for all populations, particularly menopausal women who may be vulnerable to both physical and psychological health issues (Lesser et al. 2020; Carriedo et al. 2020; Serra et al. 2022) During this period of adversity, our study focused on examining the prevalence of poor QOL among postmenopausal women living in urban Thailand amidst the COVID-19 pandemic. We found that as many as 55.85% of postmenopausal women experienced impaired QOL, as assessed using the WHOQOL-BRIEF questionnaire. Comparatively, before the pandemic, only 28.2% of peri- and postmenopausal women in Thailand reported by Panichkul, S impaired QOL, as assessed using the MENOQOL questionnaires (Panichkul et al. 2017). Interestingly, other studies reported different findings in different populations during the pandemic. For instance, Serra et al. observed no significant difference in self-report symptoms and QOL before and during the COVID-19 pandemic in Brazil. They used the Menopause Rating Scale (MRS) to evaluate menopausal symptoms and the Women’s Health Questionnaire (WHQ) to assess QOL in 341 peri- and postmenopausal women [Serra et al. 2022]. Similarly, Sievert LL et al. reported no significant differences in women’s health-related measures or physical activity during the pandemic among midlife women in western Massachusetts, United States. However, some women reported higher levels of stress and anxiety and lower levels of physical activity, while others found some benefits, such as having more time for physical activity or quality time with their families, during the pandemic (Sievert et al. 2022).

The factors associated with poor QOL during the COVID-19 pandemic were lower levels of well-being and happiness, reduced physical activity (especially for leisure activities), and menopausal symptoms, particularly exhaustion symptoms and joint pain. Previous studies have consistently linked menopausal symptoms and psychological distress, such as stress, anxiety, and depressive mood, to poor QOL in menopausal women (Panichkul et al. 2017; Chen 2020; Choijamts et al. 2022; Souza Guerra et al. 2019; Barati et al. 2021 ) Similarly, Zhang Y et al. found that the COVID-19 pandemic significantly affected mental health among residents in Liaoning Province, China, with 52% of participants reporting feelings of horror and apprehension due to the pandemic. The pandemic also affected physical activity (Zhang Y et al. 2020). In a 2022 systematic review from 8 countries, including China, Spain, Italy, Iran, the U.S., Turkey, Nepal, and Denmark, relatively high rates of anxiety (6.33–50.9%), depression (14.6–48.3%), post-traumatic stress disorder (7–53.8%), psychological distress (34.43–38%), and stress (8.1–81.9%) were reported in the general population during the COVID-19 pandemic. Female gender, younger age group (≤ 40 years), presence of chronic/psychiatric illnesses, unemployment, student status, and frequent exposure to COVID-19-related news on social media were identified as associated risk factors (Xiong et al. 2020).

Regarding physical activity during the COVID-19 pandemic, we observed a significant difference in QOL among inactive postmenopausal women who exercised more frequently (3 times per week). Carriedo et al. similarly reported that older adults who engaged in regular vigorous and moderate-vigorous physical activity during the quarantine in Spain had higher resilience scores (locus, self-efficacy, and optimism), positive affect, and lower depressive symptoms. Additionally, peri- and postmenopausal women who engaged in physical and sexual activity during the mandatory COVID-19 confinement reported higher health-related quality of life (HRQoL) and levels of resilience, while women using antidepressants had lower resilience levels (Coronado et al. 2021). HRQoL was also better in women living with others (Carriedo et al. 2020). Similarly, Lesser IA et al. found that even among inactive populations, spending more time engaged in outdoor physical activity was associated with lower anxiety than spending less time outdoors (Lesser et al. 2020). Physical activity is known to reduce menopausal symptoms, and well-established relaxation techniques and exercise can improve cardio-pulmonary and metabolic function, mental health, and overall quality of life, all impacted by menopausal changes (Nguyen et al. 2020; Mikkelsen et al. 2017).

Our study identified several factors associated with better QOL during the COVID-19 pandemic, including early menopausal age, higher education, and occasional social alcohol consumption. These findings align with a study in Iran, which reported negative correlations between QOL and the duration of menopause and positive correlations with education level in postmenopausal women (Nazarpour et al. 2020). The better QOL observed in early menopause could be attributed to higher health awareness and the earlier decline of climacteric symptoms compared to the natural onset of menopause. As for occasional social alcohol consumption, a recent systematic review reported increased frequency, quantity, and severity of alcohol use in specific segments of the population in certain countries during the early stages of the COVID-19 pandemic. While most individuals cited stress and relaxation as reasons for increased alcohol consumption, this behavior was associated with vulnerability to psychosocial distress, anxiety, depression, and overall poor mental health in some groups (Tudehope et al. 2022; Schmidt et al. 2021). In our study, we hypothesize that postmenopausal women may have turned to alcohol as a means of relaxation during stressful and challenging times. However, we do not recommend alcohol consumption as a way to improve QOL among postmenopausal women.

This report provides early insights into the QOL of postmenopausal women during the COVID-19 pandemic in Thailand. The study evaluated various factors, including socioeconomic status, general health, menopausal symptoms, COVID-19 experiences, and mental and physical activity. The QOL of postmenopausal women is influenced by mental health, well-being, physical activity, and menopausal symptoms. During the COVID-19 pandemic, poor QOL was observed in more women than before (55.85% vs. 28%). Our limitations include the fact that this study represents the impact of the COVID-19 pandemic on quality of life (QOL) in menopausal women, focusing specifically on a tertiary hospital in urban Thailand at a single time point as a cross-sectional study. Longitudinal research could provide valuable insights by comparing QOL before and after the pandemic and across different stages of reproductive aging. This would enhance our understanding of the pandemic’s long-term impact on women’s health. Our findings underscore the importance of a holistic approach to menopausal care, including attention to physical health, physical activity, mental health, well-being, and targeted menopausal symptom management.

## Conclusions

The study has revealed that postmenopausal women experienced a high prevalence of poor QOL (55.85%) during the COVID-19 pandemic, which is significantly associated with menopausal age, menopausal symptoms, education, well-being, physical activity, and alcohol consumption. It is crucial to implement interventions and strategies to improve the overall quality of life of menopausal women during the pandemic and beyond.

## Data Availability

Data available on request from the authors.
